# The antihypertensive felodipine shows synergistic activity with immune checkpoint blockade and inhibits tumor growth via NFAT1 in LUSC

**DOI:** 10.1515/med-2023-0801

**Published:** 2023-09-22

**Authors:** Si-Yu Liang, Hong-Kai Xiao

**Affiliations:** Department of Cardiology, The Fourth Affiliated Hospital of Guangzhou Medical University, Zengcheng, Guangzhou, China

**Keywords:** felodipine, antihypertensive, LUSC, ICBs

## Abstract

This study aimed to explore the role and mechanism of felodipine in lung cancer therapy. Murine subcutaneous lung squamous cancer (LUSC) models constructed by KLN-205 cells were utilized to assess the effect of felodipine monotherapy and in combination with the programmed cell death protein 1 antibody (PD1ab) and cytotoxic T lymphocyte-associated antigen-4 (CTLA4ab). Immunohistochemistry analysis was subsequently applied to detect the number of CD8+ T cells and Ki67+ cells. Lastly, a series of *in vitro* and *in vivo* experiments were performed to evaluate the effects of felodipine on human LUSC cells and explore the preliminary mechanism underlying felodipine inhibition. The results revealed that felodipine monotherapy exerted a significant inhibitory effect on LUSC growth and synergistic antitumoral activity with PD1ab and CTLA4ab. Meanwhile, immunohistochemistry analysis displayed that felodipine promoted CD8+ T-cell infiltration and downregulated Ki67 expression in tumor cells. Moreover, *in vitro* and *in vivo* experiments utilizing human LUSC cells determined that felodipine impaired the proliferative and migratory abilities of cancer cells. In addition, TCGA data analysis uncovered that nuclear factor of activated T cell (NFAT1) expression was positively correlated with overall survival and disease-free survival. Finally, the cell counting kit-8 assay signaled that felodipine might suppress tumor growth by modulating NFAT1.

## 1 Introduction

Felodipine, a member of the dihydropyridine class of calcium channel blockers (CCBs), is a first-line drug that has been extensively used for the management and treatment of essential hypertension [1]. Besides hypertension, it is widely administered for the treatment of other diseases, such as Prinzmetal angina and chronic stable angina pectoris [2]. Literature on the potential anti-tumorigenic properties of CCBs, such as verapamil and nifedipine, is scarce. A growing body of evidence suggested that the former could restrain tumor-malignant biological behavior and mitigate cancer-related mortality [3,4], whereas dissenting opinions hypothesize that the latter could increase the risk of several cancers [5,6,7]. Nifedipine has been reported to suppress colorectal cancer (CRC) progression and immune escape [8], although contrasting studies have revealed that it stimulated the proliferation and migration of different breast cancer cells via distinct pathways [9]. As for the commonly prescribed CCB, felodipine, studies on its anti-cancer activity are limited. Interestingly, a prior study reported that felodipine could inhibit cholangiocarcinoma progression and enhance the therapeutic effect of gemcitabine in nude mice [10]. However, its role and clinical significance in cancer therapy, such as in combination with immune checkpoint blockades (ICBs) for the treatment of lung cancer, remains to be elucidated.

Lung cancer remains the most lethal malignant tumor worldwide. According to a recent epidemiological investigation, an estimated 1,796,144 deaths have been attributed to lung cancer, accounting for 18% of all cancer-associated deaths globally in 2020 [11,12]. Among the subtypes of lung cancer characterized by a deficiency of known driver genes, late diagnosis, high heterogeneity, and lung squamous cell carcinoma (LUSC) occupy commonplace and are often associated with a poor prognosis. Indeed, almost half of LUSC patients have already progressed to late-stage cancer at the time of diagnosis. The 5-year survival rates of LUSC patients with stages II, III, and IV disease are approximately 32, 13, and 2%, respectively [13]. Currently, ICB therapies have emerged as the gold standard for various tumors, with programmed death 1 (PD-1), programmed death ligand 1 (PD-L1), and cytotoxic T lymphocyte antigen 4 (CTLA-4) inhibitors being the most commonly used inhibitors [14,15]. As is well documented, immune surveillance is essential for maintaining cellular homeostasis and preventing carcinogenesis [16]. Overexpression of immune checkpoint molecules such as PD-L1 and CTLA-4 in tumors can contribute to the formation of an immunosuppressive microenvironment that facilitates carcinogenesis. As a result, blockade of the PD-1/PD-L1 axis and CTLA4/B7 can eliminate these knock-on effects and remains the most common and effective checkpoint inhibition strategy. To date, many checkpoint blockade drugs have been licensed for the clinical treatment of cancer with improved overall survival (OS) time and a lower incidence of toxic side effects than traditional chemotherapeutic regimens [17]. As for LUSC patients, ICBs, such as PD-L1/PD-1 inhibitors, have significantly improved their prognosis, especially for late-stage cancer patients limited by a lack of treatment options. However, only 30% of the patients are responsive to the therapy [18,19]. Indeed, there is an urgent need to identify novel and effective functions from safe, widely clinically used drugs and develop combined therapeutic strategies with ICBs for LUSC patients.

Overall, the role of felodipine in cancer therapy, such as in combination with ICBs for the treatment of lung cancer, remains largely unknown. Herein, in this study, we aimed to investigate the action and mechanism of felodipine in LUSC tumor progression and ICB therapy.

## 2 Materials and methods

### 2.1 Cell culture and RNA interference

The murine LUSC cell line KLN-205 was purchased from Kangbai Biotechnology (Cat # CBP60080). The human LUSC cell lines SKMES-1 and NCIH226 were procured from Pricella, Wuhan, China (Cat # CL-0213, Cat # CL-0396) and American Type Culture Collection, respectively. The cells were cultured in an Eagle’s Minimum Essential Medium (Cat # M6074, MEM, Sigma-Aldrich, USA) supplemented with 10% fetal bovine serum (Cat # 10091148, Gibco) at 37°C with 5% CO_2_. All the cell lines were tested for mycoplasma, and the results were negative. When the cell confluency reached 70–80%, pancreatin was added for digestion. Human siRNA was used to knock down nuclear factor of activated T cell (NFAT1) expression, and the sequences were as follows: CCGAGTCCAAAGTTGTGTTTA (Shanghai Genechem Co., Ltd). SKMES-1 and NCIH226 were transfected with the aforementioned siRNA utilizing Lipofectamine 2000 (ThermoFisher).

### 2.2 Animal study

All animal procedures were conducted in accordance with the recommendations of the National Institutes of Health’s guidance for the use and care of laboratory animals and were approved by the Animal Care and Use Committee of Taizhou University, the ethical approval number is TZXY-2023-20231056.

DBA/2 mice (female, aged 5–6 weeks, 18–20 g) and BALB/c nude mice (female, aged 4–5 weeks, 17–19 g) were procured from the Guangdong Medical Laboratory Animal Center. 0.5–1 × 10^6^ KLN-205 cells were administered to DBA/2 mice, whereas 1–5 × 10^6^ SKMES-1 cells were administered to BALB/c nude mice. When the tumor was palpable, to establish the KLN-205 subcutaneous tumor model, 64 DBA/2 mice were randomized to the 8 following treatment groups and received retro-orbital injections of the designated drugs: control group (*n* = 8, 2 groups, PBS, intraperitoneally [i.p.], injected once every 2 days), felodipine group (*n* = 8, 2 groups, Cat # HY-B0309, MedChemExpress, 20 mg/kg, i.p., injected once every 2 days), programmed cell death protein 1 antibody (PD1ab) group (*n* = 8, 1 group, Cat # BE0146, 0.2 mg/mouse, i.p., injected once every 2 days), CTLA4ab group (*n* = 8, 1 group, Cat # BE0164, 0.1 mg/mouse, i.p., injected once every 2 days), PD1ab + felodipine group (*n* = 8, 1 group, PD1ab and felodipine) and CTLA4ab + felodipine group (*n* = 8, 1 group, CTLA4ab and felodipine). To construct the SKMES-1 subcutaneous tumor model, 16 BALB/c nude mice were randomized to the 2 following treatment groups and received retro-orbital injections of the designated drug: control group (*n* = 8, 2 groups, PBS) and felodipine group (*n* = 8, 2 groups, Cat#HY-B0309, MedChemExpress, 20 mg/kg, i.p., injected once every 2 days). The length (*a*) and width (*b*) of the tumor was measured by a slide caliper every other day, and tumor volume was calculated with the following formula: volume = *a* × *b* ^2^/2. At the same time, the weight of the mice was measured every other day. Tumor tissues were harvested when the mass reached 1,000 mm^3^ or was evidently ulcerated; cachexia occurred approximately 2–3 weeks after cell injection. The weight of the tumor was also measured. The tissues were then excised and placed in 10% neutral buffered formalin for at least 24 h. For OS analysis, another group of 80 DBA/2 mice were randomized to the 8 following treatment groups and received retro-orbital injections of the designated drug as follows: control group (*n* = 10, 2 groups, PBS), felodipine group (*n* = 8, 2 groups), PD1ab group (*n* = 8, 1 group), CTLA4ab group (*n* = 8, 1 group), PD1ab + felodipine group (*n* = 8, 1 group, PD1ab and felodipine), and CTLA4ab + felodipine group (*n* = 8, 1 group, CTLA4ab and felodipine). The endpoint was defined as follows: the tumor volume attained 1,500 mm^3^, or the diameter of the ulcer exceeded 1.5 cm. The procedure references the animal study of a recent report [8,20].

### 2.3 Immunohistochemistry

The tissues of each group (control group, felodipine group, PD1ab group, PD1ab + felodipine group) were excised and placed in 10% neutral buffered formalin for the same treatments as follows. After the sections of each group (control group, felodipine group, PD1ab group, PD1ab + felodipine group) suffer baking and dewaxing, the sections received same treatments as follows. IHC analysis was conducted using a kit (Cat # K135925C, ZSGBBIO, Beijing, China). After incubating with primary antibodies against CD8 (Cat # ab209775, ABCAM, 1:1,000) and Ki67 (Cat#ZM-0167, ZSGB-BIO, 1:400) and staining with 3,3-diaminobenzi-dine (DAB) and Mayer’s hematoxylin, the sections were photographed, and the number of positive cells was counted. The procedure references the material and method used in a recent literature [21].

### 2.4 Cell counting kit-8 (CCK-8)

Cell proliferation was analyzed using a CCK-8 (Cat # CK04, Dojindo, Japan). KLN-205 and SKMES-1 NICH226A cells were plated in 96-well plates at a density of 1,000 cells/well. After adding felodipine, cell proliferation of each group (DMSO group, felodipine [10 μM] group, felodipine [50 μM] group) was tested on days 0, 1, 2, 3, 4, 5, and 6 after adding CCK-8 reagent utilizing a microplate reader (Cat# 1681135, Bio-Rad Laboratories Inc, USA), and absorbance was measured at 450 nm. This assay references the material and method used in a recent report [22].

### 2.5 Colony formation assay

SKMES-1 cells were plated in 6-well plates with felodipine at a density of 400 cells/well at 37°C and 5% CO_2_ for 10–14 days. Each group (DMSO group, felodipine [10 μM] group) received same treatments as follows. The medium consisted of Eagle’s Minimum Essential Medium supplemented with 10% fetal bovine serum and was timely replaced. When the cell colonies were visible, they were fixed and stained with 0.1% crystal violet. Afterward, the plates were photographed, and the cell colonies were counted. This experiment references the material and method used in a recent report [21].

### 2.6 Wound healing assay

Cells were plated in 6-well plates at a density of 5 × 10^5^ SKMES-1 cells/well. When the cells approached 100% confluency, a 10 μL sterile pipette tip was employed to scratch a straight line. At the same time, felodipine was added to the medium. Each group (DMSO group, felodipine [10 μM] group) received same treatments as follows. The shapes of the straight lines were photographed at specified time points (0, 12, 24, and 36 h). This experiment was followed by the material and method in a recent report [23].

### 2.7 Real-time quantitative PCR (qPCR)

SKMES-1 and NCIH226 cells were plated in 6-well plates at a density of 5 × 10^5^ cells/well and cultured in a medium containing felodipine. Each group (DMSO group, felodipine [10 μM] group, felodipine [50 μM] group, felodipine [100 μM] group) received same treatments as follows. After 48 h, the cells were collected, and the total RNA was extracted with a TRIzol reagent (Invitrogen, USA). cDNA was reverse-transcribed using a Prime-Script RT reagent Kit (Promega, Madison, WI, USA). The SYBR Premix EX Taq™ (Takala, Dalian, China), operating on an ABI 7500 Real-Time PCR system (Applied Biosystems, Foster City, USA), was used for qPCR. The primer sequences for NFAT1 amplification were as follows: 5′-CGATTCGGAGAGCCGGATAG-3′ (forward) and 5′-TGGGACGGAGTGATCT CGAT-3′ (reverse) (synthesized by Shenggong Biotechnology, Guangzhou, China). GAPDH served as an internal control. Relative gene expression was calculated by the comparative 2^−ΔΔCT^ method. The procedure follows the instruction of test kits and references the material and method used in a recent report [24].

### 2.8 Statistical analysis

Soft EXCEL was utilized to collect the data. GraphPad 9.02 was used to perform statistical analyses. Continuous variables with normal distribution and non-normal distribution were expressed as mean ± standard deviation (SD) and median (interquartile range), respectively. The Student’s *t*-test (two-tailed) was used for group comparison. The survival rates were evaluated by Kaplan–Meier method and tested by log-rank test. *p* < 0.05 was considered statistically significant.

## 3 Results

### 3.1 Felodipine suppressed LUSC growth and promoted tumor immune responses to ICBs

**Figure 1:**
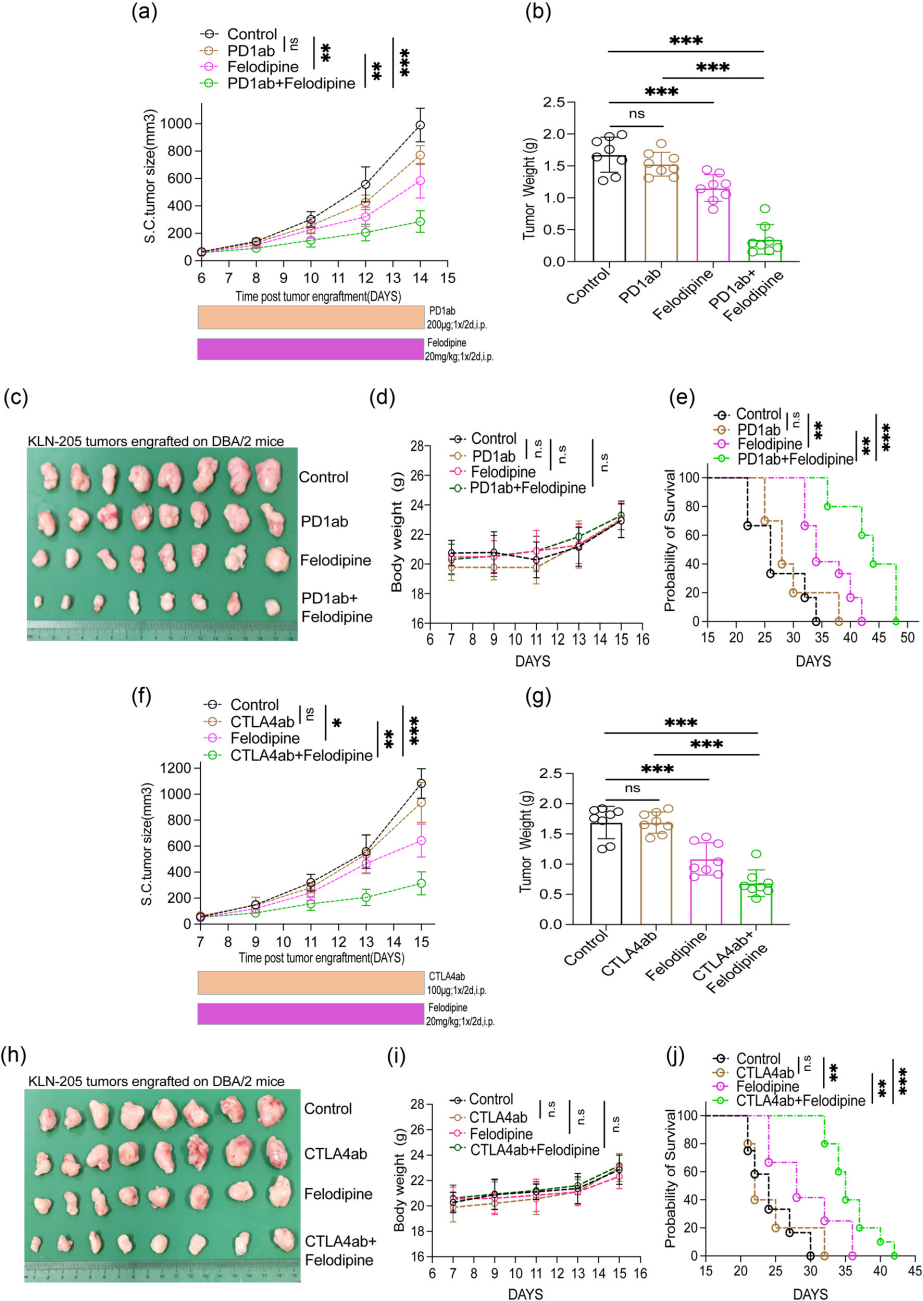
Felodipine suppressed LUSC growth and strengthened tumor immune responses to ICBs. (a–c) Felodipine plus PD1ab inhibited KLN-205 tumor growth in DBA/2 mice (*n* = 8). (d) The weight of mice. (e) The survival time of mice receiving felodipine plus PD1ab (*n* = 10). (f)–(h) Felodipine plus CTLA4ab inhibited KLN-205 tumor growth in DBA/2 mice (*n* = 8). (i) The weight of mice. (j) The survival time of mice receiving felodipine plus CTLA4ab (*n* = 10). Data are presented as mean ± SD, n.s. no significance; *p < 0.05, **p < 0.01, ***p < 0.001, ****p < 0.0001. Error bars denote s.e.m.

Felodipine is a dihydropyridine calcium-channel antagonist that significantly reduces diastolic and systolic blood pressure in hypertensive patients and exerts beneficial hemodynamic effects in patients with congestive heart failure and chronic stable angina pectoris. Herein, analyzing the subcutaneous tumor model in immunocompetent DAB/2 mice exposed that felodipine monotherapy significantly inhibited the grafted KLN-205 cells growth, whereas PD1ab monotherapy exerted no significant inhibitory effect compared with the control group. Surprisingly, felodipine plus PD1ab improved the inhibitory effects compared with PD1ab monotherapy, indicating that felodipine may potentiate tumor immune responses to PD1ab (Figure 1a–c). When compared with the control group, the body weight of mice in each group (felodipine, PD1ab, and combination group) showed no significant change (Figure 1d). Furthermore, the survival time of mice in the felodipine monotherapy group and the felodipine plus PD1ab group was significantly longer than the control group and PD1ab monotherapy group, respectively (Figure 1e). Similar results were observed upon the administration of CTLA4ab and felodipine in the tumor-bearing mice. More specifically, tumor growth was slower in the KLN205 tumors of mice in the felodipine monotherapy and the felodipine combined with CTLA4ab groups compared with that of the control and CTLA4ab monotherapy groups (Figure 1f–h). When compared with the control group, the body weight of mice in each group (felodipine, PD1ab, and combination group) showed no significant change (Figure 1i). Consequently, mice in the felodipine monotherapy group and those in the felodipine plus CTLA4ab group achieved longer survival outcomes than those in the control group and the CTLA4ab monotherapy group (Figure 1j).

**Figure 2:**
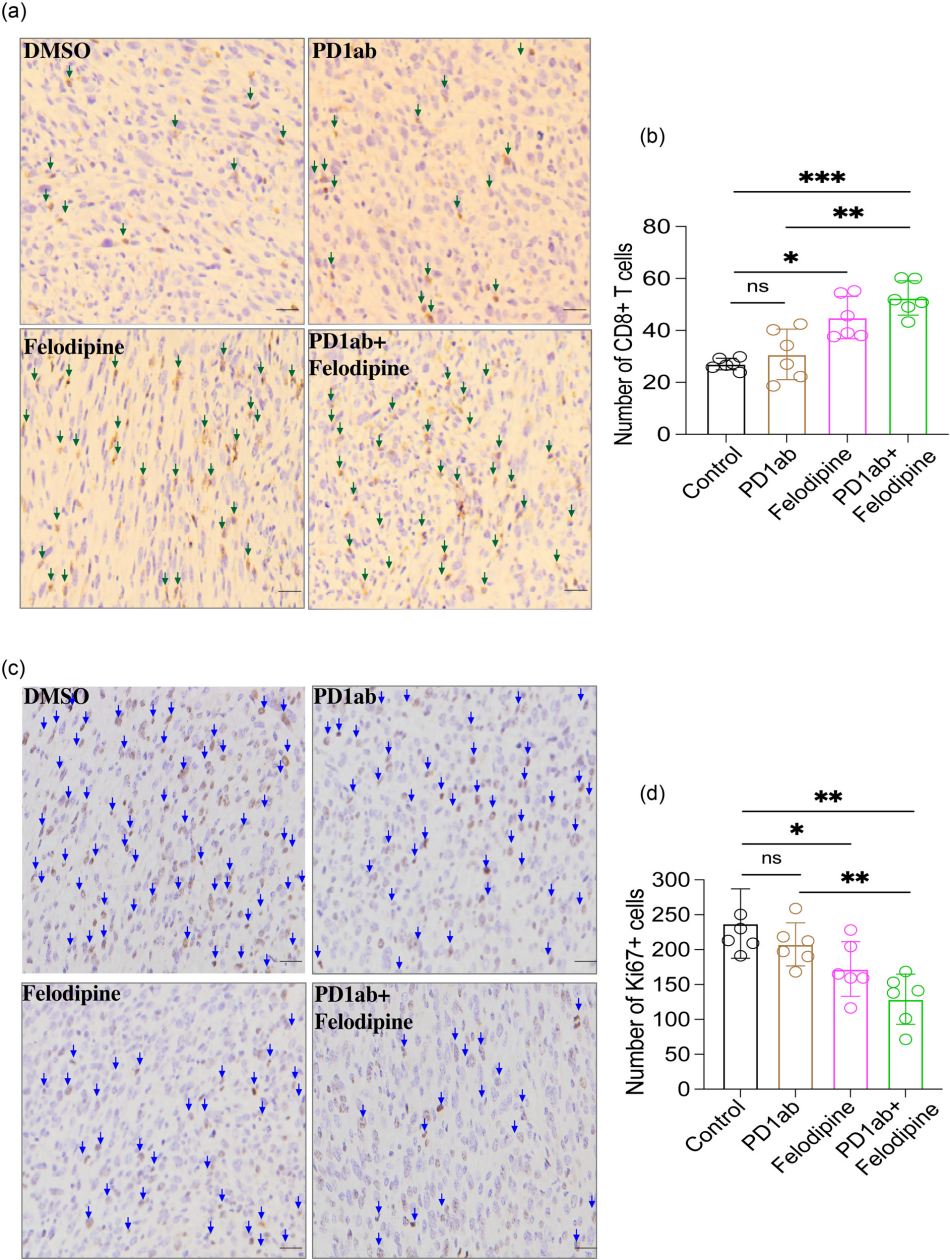
IHC analysis of the tumor tissue after receiving felodipine plus PD1ab treatment. (a and b) IHC analysis of CD8+ T-cell infiltration of KLN-205 tumor tissue after treatment, representative positive cells were marked with arrows; (c and d) IHC staining of Ki67+ cell infiltration of KLN-205 tumor tissue after treatment, representative positive cells were marked with arrows; scale bars, 20 μm. Data are expressed as mean ± SD, n.s. no significance; *p < 0.05, **p < 0.01, ***p < 0.001, ****p < 0.0001. Error bars denote s.e.m.

IHC detection of the KLN-205 tumors revealed that felodipine promoted CD8+ T-cell infiltration and decreased Ki67 expression compared with the control group. Besides, an increased number of CD8+ T cells as well as a decreased Ki67 expression were noted in the tumor microenvironment of the felodipine plus PD1ab group (Figure 2a–d).

### 3.2 Felodipine inhibited human LUSC proliferation and migration

**Figure 3:**
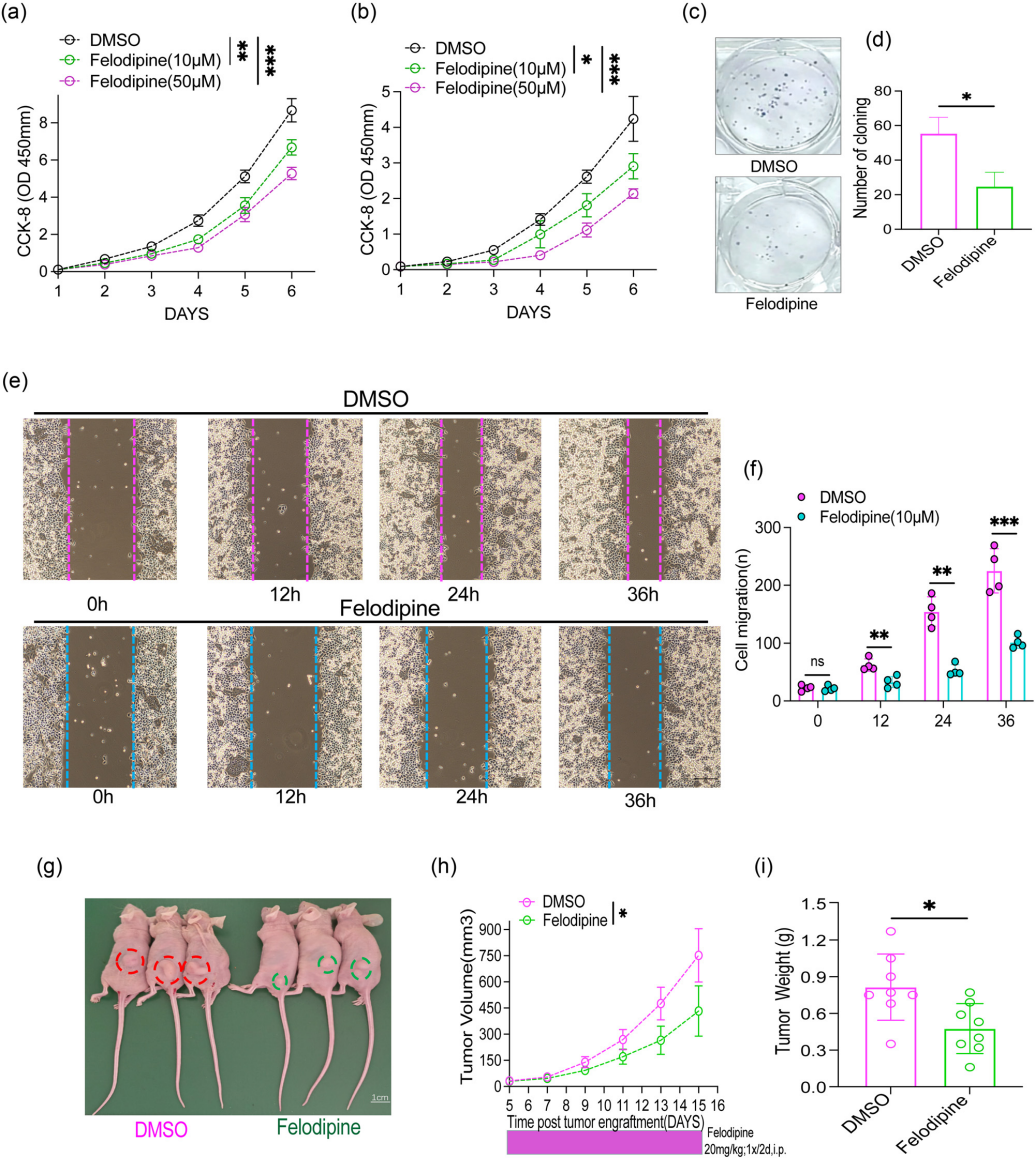
Felodipine inhibited human LUSC proliferation and migration. (a) CCK-8 assay analyzing SKMES-1 proliferation after felodipine (0, 10, 50 μM) treatment. (b) CCK-8 assay investigating NCIH226 proliferation after felodipine (0, 10, 50 μM) treatment. (c and d) Colony formation assay evaluating SKMES-1 proliferation after felodipine (10 μM) treatment. (e and f) Wound healing assay examining SKMES-1 migration after felodipine (10 μM) treatment; scale bars, 200 μm. (g) – (i) Subcutaneous tumor model in nude mice (*n* = 8) evaluating SKMES-1 growth following felodipine treatment (20 mg/kg); data are expressed as mean ± SD, n.s. no significance; *p < 0.05, **p < 0.01, ***p < 0.001, ****p < 0.0001. Error bars denote s.e.m.

The impact of felodipine on the proliferative ability of the SKME-1 and NCIH226 human LUSC cell lines was investigated using CCK-8 and colony formation assays. The results of the CCK-8 assay indicated that felodipine significantly impaired the proliferative ability of both SKME-1 and NCIH226 cells (Figure 3a and b). Moreover, the inhibitory effect of felodipine was validated by the colony formation assay (Figure 3c and d). Similarly, the migratory capability of SKME-1 human LUSC cells was significantly suppressed by felodipine (Figure 3e and f). Nude mice were subcutaneously injected with human LUSC SKME-1 cells to verify the effects of felodipine *in vivo*, and the results demonstrated that tumor tissues of mice receiving felodipine were smaller and lighter than those in the control group, suggesting that felodipine suppressed LUSC proliferation *in vivo* (Figure 3g–i).

### 3.3 Felodipine suppressed human LUSC progression via NFAT1

**Figure 4:**
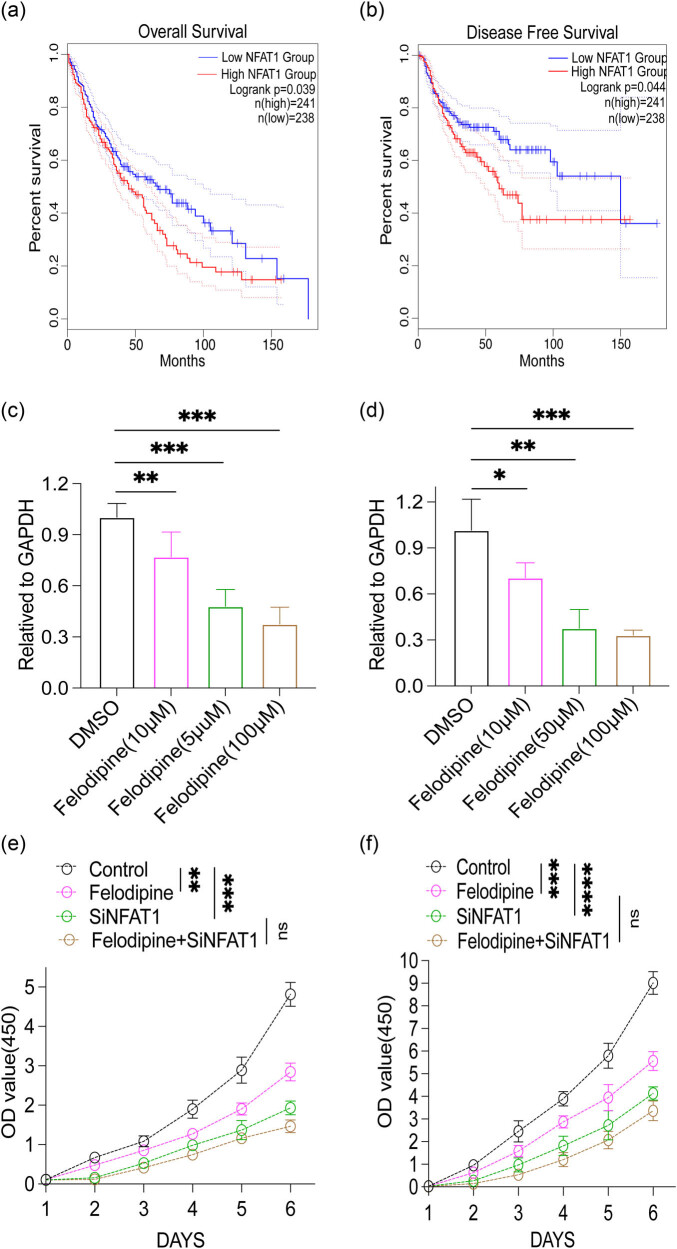
Felodipine suppressed human LUSC progression via NFAT1. (a and b) Data from the TCGA database were used to explore the relationship between OS, DFS, and NFAT1 expression in LUSC patients, respectively. (c) qPCR detection of NFAT1 after felodipine (10, 50, 100 μM) treatment in SKMES-1. (d) qPCR detection of NFAT1 after felodipine (10, 50, 100 μM) treatment in NCH226. (e) CCK-8 assay examining SKMES-1 proliferation after felodipine treatment and NFAT1 knockdown. (f) CCK-8 assay examining NCH226 proliferation after felodipine treatment and NFAT1 knockdown. Data are presented as mean ± SD, n.s. no significance; *p < 0.05, **p < 0.01, ***p < 0.001, ****p < 0.0001. Error bars denote s.e.m.

TCGA data analysis determined that upregulation of NFAT1 was negatively correlated with OS and disease-free survival (DFS) in LUSC patients (Figure 4a and b), signifying that the clinical significance of NFAT1 in LUSC cannot be overlooked. Additionally, qPCR analysis demonstrated that felodipine significantly downregulated NFAT1 expression in SKME-1 and NCIH226 human LUSC cells (Figure 4c and d). Furthermore, the CCK-8 assay showed that felodipine and NFAT1 knockdown by RNA interference technology significantly decreased the proliferative ability of SKMES-1 cells compared with the control group. Nevertheless, there was no significant difference in the felodipine plus si-NFAT1 group compared with the si-NFAT1 group (Figure 4e); that is to say, felodipine may lose its inhibitory effect after NFAT1 knockdown. Consistent results were observed by utilizing another human LUSC cell line, namely NCIH226 (Figure 4f). Nonetheless, further experiments and mechanistic exploration are warranted to validate the credibility of our results.

## 4 Discussion

In this study, felodipine showed synergistic activity with ICBs, including PD1ab and CTLA4ab, and suppressed the progression of LUSC by inhibiting cell proliferation and migration by regulating NFAT1. This is the first study to investigate the action and mechanism of felodipine in LUSC *in vivo* and *in vitro*. Collectively, these findings indicated that felodipine can be used for clinical treatment of LUSC.

Given the high incidence of hypertension and lung cancer, both diseases are frequently co-diagnosed since they share common denominators, such as the age of onset. Besides, a large body of evidence insinuated that hypertension is closely correlated with the incidence and prognosis of common malignant tumors, including lung, colon, oral, esophageal, and laryngeal cancers [25,26,27,28,29]. Cancer patients with hypertension typically have a worse prognosis than normotensive ones [30,31]. Therefore, it is vital to identify the role of common first-line antihypertensives such as felodipine in cancer treatment. The research here has at least three advantages: (1) it affirms the position of felodipine as the drug of choice in cancer patients with comorbid hypertension, Prinzmetal’s variant angina, and chronic stable angina pectoris undergoing cancer treatment, especially immunotherapy. (2) the study may offer novel insights into the development of therapeutic strategies for the treatment of cancer using commonly prescribed drugs. (3) it is conducive to assuring the safety of felodipine for cancer prevention.

Felodipine is a first-line antihypertensive that belongs to the dihydropyridine class of CCBs. CCBs such as verapamil and nifedipine have been reported to exert anti-tumorigenic effects in various cancers, including CRC, skin cancer, and lung cancer. While previous studies predominantly focused on cancer stemness and chemotherapy resistance [32,33,34,35,36], there is a paucity of studies on ICBs such as CTLA4ab, which suppresses expression of CTLA4 to promote proliferation of T cell to attack the tumor cells and are also the standard of care, either as monotherapy or in combination with other drugs for lung cancer therapy [37], however, a considerable portion of patients do not benefit from it. Indeed, report about what effect of CCBs including verapamil, nifedipine and felodipine will generate on this ICBs is rare. As for another common ICBs PD1ab, only one study reported that nifedipine could suppress CRC progression and immune escape by mitigating NFAT2 nuclear translocation, thereby enhancing the effect of PD1ab on tumor inhibition [8]. However, it is worthwhile emphasizing that the pharmacological properties of these three CCBs are distinct. Compared with verapamil and nifedipine, felodipine has a longer duration of action and wider application range in clinical practice [38]; yet its role in LUSC treatment remains unknown, especially when used in conjunction with ICB therapy. In our research, felodipine was verified to show synergistic activity with ICBs including PD1ab and CTLA4ab. And felodipine also significantly increased the infiltration of CD8 + T cells into the tumors. In view of reports about PD1ab and CTLA4ab combination [39,40,41], we may hypothesize that felodipine alter the vascular permeablility for immune cells or induce cytokines secretion and reprogram the tumor immune microenvironment to an suppressed status for enhancing the tumor inhibition of PD1ab and CTLA4ab. The detailed mechanism needs further study.

Laboratory research on felodipine in tumor prevention is scarce. Related research described that felodipine could be repurposed to target the TRPV1 receptor and relieve oral cancer pain [42]. Another study evinced that felodipine could directly suppress cholangiocarcinoma progression and potentiate the therapeutic effect of gemcitabine *in vivo* [10]. Therefore, this research employed the human LUSC cell lines SKMES-1 and NCIH226 to demonstrate that felodipine can suppress proliferative and migratory abilities both *in vivo* and *in vitro*. Nevertheless, research on its inhibitory effects in cholangiocarcinoma is limited to phenotypic studies rather than mechanistic studies. Again, recent research concluded that nifedipine inhibited CRC progression by modulating NFAT2, which may provide insights into novel targets of felodipine for tumor inhibition and immune response to PD1ab.

Calcium-dependent NFAT is a vital transcription family involved in mediating tumor development, which governs angiogenesis, homeostasis, inflammatory response, and the immune system in bones [41,42,43]. Emerging evidence indicates that the NFAT family remains activated and participates in the progression of numerous cancers, such as non-small cell lung cancer, pancreatic cancer, CRC, and breast cancer, thereby playing a decisive role in the malignant biological behaviors of tumors [43,44,45]. Furthermore, a recent study reported that another common calcium-channel antagonist, namely nifedipine, suppressed CRC progression by modulating NFAT2 [8]. Nevertheless, the TCGA database identified no significant correlation between NFAT2 expression and the prognosis of LUSC patients (data not shown). Naturally, the function of NFAT in LUSC is poorly understood. As another important member of the NFAT family, NFAT1 plays a role in the progression of several tumors, including breast cancer, renal cell carcinoma, melanoma, and glioma [46,47,48,49]. Yet, its specific role in tumor immunotherapy and the progression of LUSC is elusive. This study noted that the poor prognosis of LUSC patients was closely associated with high expression of NFAT1. Meanwhile, felodipine can significantly down-regulate the expression of NFAT1 and further suppress SKMES-1 and NCIH226 cell proliferation. Conversely, NFAT1 knockdown reversed the effect of felodipine on tumor inhibition compared with the control group. In other words, the inhibitory effect of felodipine may potentially be NFAT1-dependent.

## 5 Conclusion

**Figure 5:**
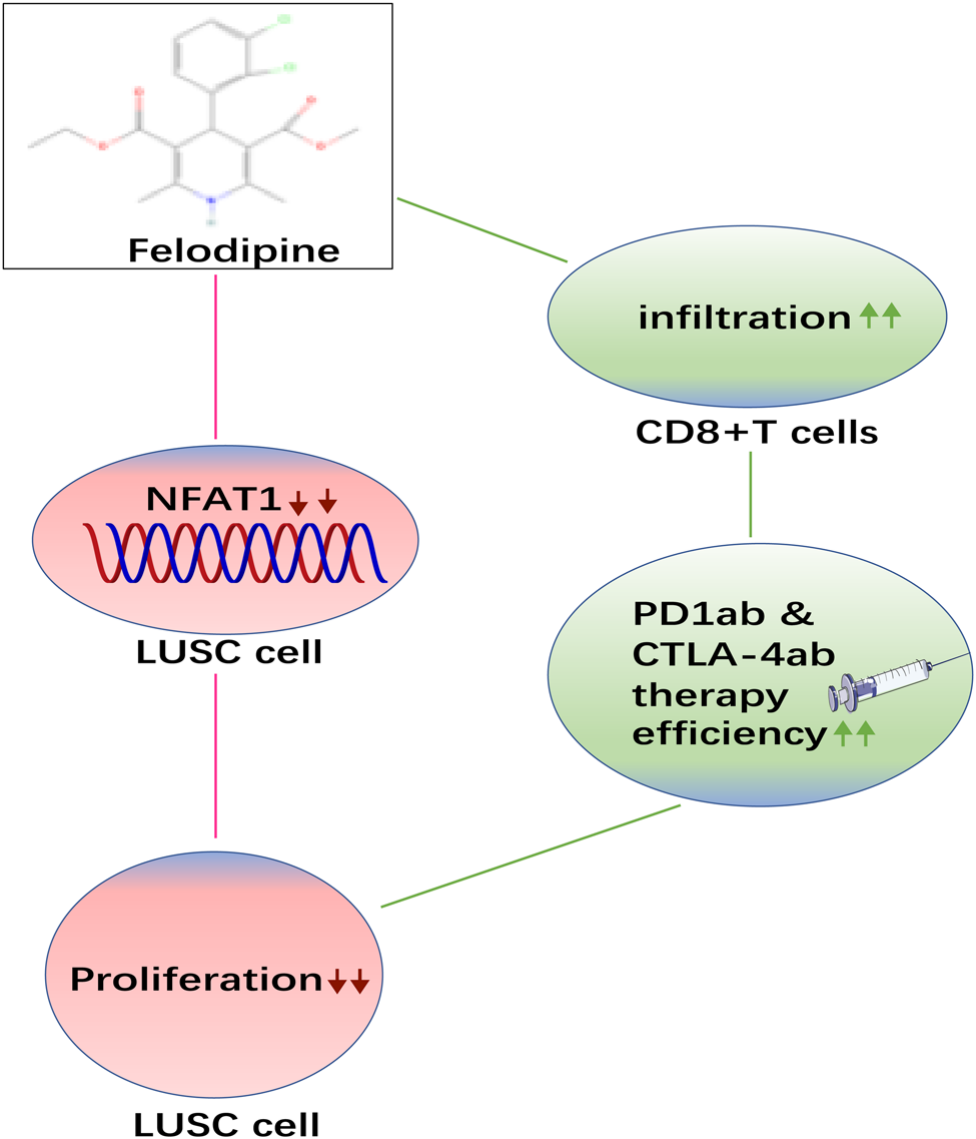
A schematic diagram illustrating felodipine exhibited synergistic activity with ICB and inhibited tumor growth by mediating NFAT1 expression in LUSC.

To the best of our knowledge, this is the first study to investigate the action and mechanism of felodipine in LUSC *in vivo* and *in vitro*. Taken together, felodipine exhibited synergistic activity with ICB and inhibited tumor growth by mediating NFAT1 expression in LUSC (Figure 5). Our research provides elementary experimental results for the treatment of LUSC using felodipine, but there are still some limitations that cannot be overlooked such as more direct evidence are warranted to verify that felodipine inhibited tumor growth via NFAT1, in consideration of the time and cost of the trial, we can only explore this mechanism by simple and feasible experiments of the present kind. More experiments *in vivo* and *vitro* are needed to validate the results of this study. We will explore this in more depth in the next stage.
